# Decoding semantic sound categories in early visual cortex

**DOI:** 10.1093/cercor/bhaf208

**Published:** 2025-08-02

**Authors:** Giusi Pollicina, Samuel A Müller, Polly Dalton, Petra Vetter

**Affiliations:** Department of Psychology, Royal Holloway, University of London, Egham Hill, Egham, Surrey, TW20 0EX, United Kingdom; Department of Psychology, Birkbeck, University of London, Malet Street, Bloomsbury, London, London WC1E 7HX, United Kingdom; Department of Psychology, University of Fribourg, Rue P.-A.-de-Faucigny 2, 1700 Fribourg, Fribourg, Switzerland; Department of Psychology, Royal Holloway, University of London, Egham Hill, Egham, Surrey, TW20 0EX, United Kingdom; Department of Psychology, University of Fribourg, Rue P.-A.-de-Faucigny 2, 1700 Fribourg, Fribourg, Switzerland

**Keywords:** audition, early visual cortex, fMRI, multisensory interaction, MVPA

## Abstract

Early visual cortex, once thought to be exclusively used for visual processes, has been shown to represent auditory information in the absence of visual stimulation. However, the exact information content of these representations is still unclear, as is their degree of specificity. Here, we acquired functional magnetic resonance imaging (fMRI) data while blindfolded human participants listened to 36 natural sounds, hierarchically organized into semantic categories. Multivoxel pattern analysis revealed that animate and inanimate sounds, as well as human, animal, vehicle, and object sounds could be decoded from fMRI activity patterns in early visual regions V1, V2, and V3. Further, pairwise classification of the different sound categories demonstrated that sounds produced by humans were represented in early visual cortex more distinctively than other semantic categories. Whole-brain searchlight analysis showed that sounds could be decoded also in higher level visual and multisensory brain regions. Our findings extend our understanding of early visual cortex function beyond visual feature processing and show that semantic and categorical sound information is represented in early visual cortex, potentially used to predict visual input.

## Introduction

Early visual cortex receives different types of signals: feedforward visual input from the retina, as well as feedback information from higher level brain areas or other sensory modalities, eg, audition or touch (eg Monaco et al. 2017; Petro et al. 2017). In the absence of visual stimulation from the retina, ie, during eyes-closed conditions or in congenital blindness, natural sounds such as birds chirping, traffic noise, and people talking can be decoded from fMRI activity patterns in human early visual cortex (Vetter et al. 2014; Vetter et al. 2020). Thus, content-specific and categorical information from audition is fed back to early visual cortex and is represented there, possibly for the purpose of predicting incoming visual information (eg Bar 2007; Friston 2010; Petro et al. 2014). With a slightly different selection of sounds, successful sound decoding mostly works in early visual cortex of blind individuals (Van den Hurk et al. 2017; Mattioni et al. 2020), and it remains unexplored whether sound decoding in blindfolded sighted participants is limited to the specific sound exemplars used in previous studies. More critically, given the variety of feedback signals to early visual cortex and their importance for predictive processing (Petro et al. 2014), it is unclear which type of auditory information content is transferred via feedback all the way down to early visual cortex: are only broad semantic sound categories, such as animate and inanimate sounds (Vetter et al. 2014), or also more fine-grained semantic sound categories, eg human, animal, vehicle, and object sounds, represented in early visual cortex? Answering this question would get us closer to the semantic content of information and its level of abstraction that is transferred via feedback to early visual cortex.

Much previous audio–visual interaction research has focused on how signals from audition and vision are integrated across space and time (eg Koelewijn et al. 2010), usually using simple stimuli such as beeps and flashes or visual gratings (eg Chen et al. 2011a; Caclin et al. 2011; Chanauria et al. 2019). However, beeps and gratings do not contain ecologically valid information content, and the question of whether semantic information is communicated between audition and vision has been addressed much less. In ecologically valid environments we encounter in everyday life, the sounds that accompany our vision carry substantial information content with far-reaching consequences for our interactions with the world. For example, when we walk down a busy road and hear the sound of an approaching car from behind, our brain predicts seeing a car a moment later (eg Bar 2007; Friston 2010). When we aim to cross the street, it is critical for our survival that our brain has identified the sound behind us as that of an approaching car rather than a stationary car and has prepared for the relevant visual processing.

The current study aimed to determine which semantic categories of natural sounds are fed down to and represented in early visual cortex, and to what degree of categorical specificity. In this functional magnetic resonance imaging (fMRI) study, we presented blindfolded sighted participants with a large sample of 36 natural sounds, hierarchically organized into semantic categories at different levels of abstraction, and used multivariate pattern analyses (MVPA) to decode different sound categories from fMRI activity in individually retinotopically mapped early visual cortex regions.

**Fig. 1:**
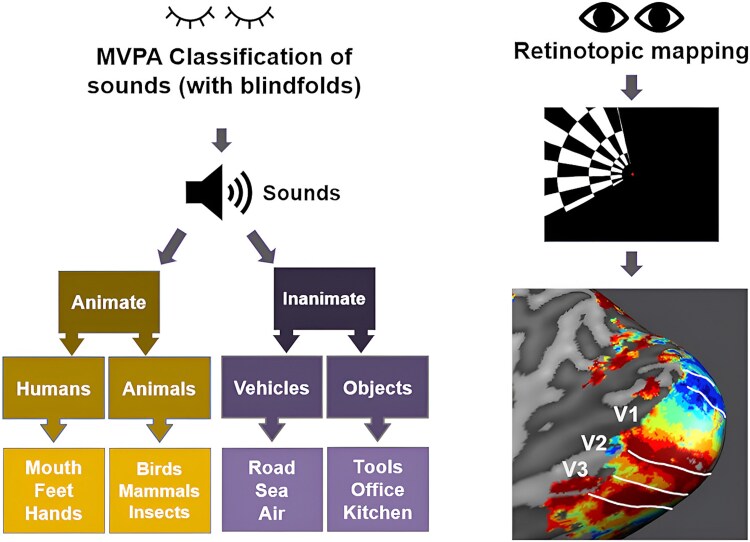
fMRI experimental design and procedure. Participants were blindfolded and attentively listened to 36 different natural sounds, subdivided into several semantic categories, while detecting an occasional target tone present in 10% of the trials. For retinotopic polar mapping of early visual cortex regions, participants watched a flickering rotating wedge and detected a color change at the center. We mapped V1, V2, and V3 in each participant using retinotopic activity maps projected onto individually reconstructed cortical surfaces. The lower right panel shows the right hemisphere retinotopic map of one example participant.

Regions higher in the hierarchy of visual processing have been extensively reported to be sensitive to different semantic categories of visual objects, with studies suggesting that the ventrotemporal cortex is structured to differentiate between animate and inanimate stimuli (eg Grill-Spector and Weiner, 2014; Bracci and de Beeck 2016; Martin et al. 2018; Blumenthal et al. 2018) and to preferentially activate for animals versus tools and vice versa with a certain degree of spatial distinction (Chao and Martin 2000; Beauchamp et al. 2002; Bola et al. 2022). Moreover, visual object stimuli belonging to the same semantic category are represented with similar patterns of neural activation in inferior temporal cortex (Kriegeskorte et al. 2008; Bracci and de Beeck 2016). We drew from this body of evidence to select the categories of our auditory stimuli: animate, subdivided into human and animal sounds, and inanimate, subdivided into vehicle and object sounds (see Fig. 1). We tested which of those semantic categories, shown to be distinguished in high-level visual cortex when presented visually, could be distinguished in early visual cortex when presented auditorily, and in the absence of visual input. This allowed us to determine the semantic information content contained in auditory feedback to early visual cortex.

## Materials and methods

### Participants

21 healthy adult volunteers were recruited for the study; all signed informed consent and were paid for their participation. A power analysis (Faul et al. 2007) based on average effect sizes found previously (0.6, Vetter et al. 2014) showed that 19 participants are sufficient to reach 80% power. The data of 3 participants were excluded due to poor sound stimuli recognition (less than 50% sound recognition accuracy) or excess head movements inside the scanner, leaving us with a sample size of 18 participants (12 females, mean age 24) resulting in 79% power. The study was approved by the Research Ethics Committee of Royal Holloway, University of London (Project ID 1191).

### Sound stimuli

We selected the natural sound stimuli based on behavioral piloting, sampling the sounds from different online databases (eg YouTube, FreeSound, and SoundBible). We selected them to fit the following criteria: (i) be as recognizable and unambiguous as possible, and (ii) not conveying any emotion or containing meaningful speech. Thus, samples of laughter and crying were excluded, as well as sounds that could easily be mistaken for something else (ie bacon sizzling in a pan was often mistaken for rain, so it was not optimal for this experiment). Based on behavioral piloting (*n* = 15), we selected sounds that were recognized with a minimum accuracy of 75%, and a mean accuracy of 90.8% (SD 8.61) in the final set of sound stimuli. The final set used in the fMRI experiment consisted of 36 natural sounds with lengths ranging from 2 to 3 s (full list of sounds and link to sound files in Table 1). These were categorized in a hierarchical fashion: inanimate and animate sounds at the highest tier; animals, humans, objects, and vehicles at the intermediate level, and further labeled with their specific subcategory in the last tier (see Fig. 1). Three sound exemplars were used for each subcategory (ie “seagull,” “duck,” and “chicken” for the “birds” category). All sounds were normalized for amplitude, equalized with custom digital equalization filters (Sensimetrics) and presented in mono to both ears. Supplementary Figs. S1 to S3 show amplitude profiles, amplitude envelopes, and spectrograms, respectively, for all 36 sounds.

**Table 1:** Full list of sound stimuli divided by category. Link to the sounds folder: https://www.dropbox.com/sh/j752a6la0upje8d/AABX8aLmdc_5wNLhrFw8kUfna?dl=0

Superordinate	Intermediate	Subordinate	Exemplars
Animate	Humans	Mouth sounds	Baby
Animate	Humans	Mouth sounds	Cough
Animate	Humans	Mouth sounds	Talking (invented language)
Animate	Humans	Feet sounds	Walking with heels
Animate	Humans	Feet sounds	Walking on gravel
Animate	Humans	Feet sounds	Marching
Animate	Humans	Hand sounds	Person clapping
Animate	Humans	Hand sounds	Audience applause
Animate	Humans	Hand sounds	Snapping fingers
Animate	Animals	Mammals	Dog
Animate	Animals	Mammals	Sheep
Animate	Animals	Mammals	Horse
Animate	Animals	Birds	Chicken
Animate	Animals	Birds	Duck
Animate	Animals	Birds	Seagull
Animate	Animals	Insects	Bee
Animate	Animals	Insects	Cricket
Animate	Animals	Insects	Mosquito
Inanimate	Vehicles	By road	Bike
Inanimate	Vehicles	By road	Car
Inanimate	Vehicles	By road	Motorbike
Inanimate	Vehicles	By air	Helicopter
Inanimate	Vehicles	By air	Plane
Inanimate	Vehicles	By air	Jet
Inanimate	Vehicles	By sea	Ferry
Inanimate	Vehicles	By sea	Tugboat
Inanimate	Vehicles	By sea	Jetski
Inanimate	Objects	Tools	Drill
Inanimate	Objects	Tools	Hammer
Inanimate	Objects	Tools	Handsaw
Inanimate	Objects	Kitchen	Microwave
Inanimate	Objects	Kitchen	Teaspoon
Inanimate	Objects	Kitchen	Soda can
Inanimate	Objects	Office	Phone
Inanimate	Objects	Office	Scissors
Inanimate	Objects	Office	Typewriter

See Supplementary Figs. S1, S2, and S3 for amplitude profiles, amplitude envelopes and spectrograms, respectively, as well as Supplementary Table S1 for individual sound recognition accuracy post-scanning.

### Data acquisition and experimental procedure

We acquired blood oxygen level dependent (BOLD) signals in a 3 T Siemens Tim Trio MRI scanner with a 32-channel phased array head coil (bandwidth 1628 Hz, TR = 2.0 s, TE = 30.6 ms, resolution 2.0 × 2.0 × 2.0 mm, 48 slices, flip angle 78°). A 6-min long T1 weighted structural MRI scan was also acquired. Participants were placed in the MRI scanner first for retinotopic mapping and then for the functional runs with sound stimulation. For retinotopic polar mapping, participants were instructed to fixate on a red cross at the center of their visual field while a black and white checkered wedge (22.5 deg wide) rotated anticlockwise across their visual field. To ensure participants kept central fixation, they were asked to press a button every time the cross at the center changed color. The run lasted about 8 min, comprising 12 cycles of wedge rotation, in line with standard retinotopic polar mapping procedures (eg Wandell et al. 2007; Muckli et al. 2009; Schira et al. 2009). After retinotopic mapping, participants were taken out of the scanner, had a short break, and were given blindfolds and in-ear headphones to wear during the functional runs with sound stimulation. Participants were blindfolded and instructed to keep their eyes closed at all times and the room lights were switched off to ensure as little feedforward visual stimulation as possible in sighted participants. Sounds were presented individually, followed by an inter-stimulus interval of 4 s (jittered at 0.5 s) and in pseudo-randomized order. Participants were asked to actively and carefully listen to the sounds. To ensure participants paid attention to all sounds, they were asked to press a button with their index finger every time they heard a “beep” noise on top of a fraction of stimuli. The beep noises were either high-pitch (800 Hz) or low-pitch (400 Hz) pure tones, present in 10% of the trials. An additional 10% of trials were null events without sound stimulation to allow brain activity to return to baseline. We used 3 sound exemplars in each of the 12 subcategories (see Fig. 1 and Table 1), adding up to 36 sound stimuli in total. Each sound was presented 12 times, in a pseudo-randomized order, totaling 432 trials, divided into 4 functional runs. To ensure that participants had identified the sounds accurately inside the scanner, participants listened to the 36 sounds once more on the lab PC at the end of the scanning session, reported the identity of the sounds, how confident they felt in their choice on a scale 1 to 10, and whether their perception matched that inside the scanner. Sounds were correctly recognized with an average of 96% accuracy (list with recognition rates of individual sounds in Supplementary Table S1), matching well the average recognition accuracy of 90.8% in the behavioral pilot. Participants also completed a Vividness of Visual Imagery Questionnaire (VVIQ; Marks 1973), which included picturing items or scenes in their minds first with eyes closed and then with eyes open and rating the vividness of their imagination on a scale of 1 to 5.

### Data analyses

Functional MRI data were pre-processed with BrainVoyager 22.2 (BrainInnovation) with standard preprocessing steps (ie slice scan time correction, temporal high-pass filter, 3D rigid body motion correction), but without spatial smoothing in the sound stimulation runs (see Gardumi et al. 2016, and Hendriks et al. 2017, for a detailed discussion on the costs and benefits of spatial smoothing in MVPA analyses). Region of interests (ROIs) for V1, V2, and V3 were defined on individual reconstructed cortical surfaces using the activation gradients from individual retinotopic polar mapping (see Fig. 1 for a retinotopic map of early visual ROIs of an example participant). V1, V2, and V3 were collated to create an additional ROI, comprising the whole early visual cortex (EVC). ROIs for auditory cortex were defined using the contrast all sounds > no sound stimulation (background MRI noise was always present). Supplementary Table S2 reports mean, minimum, and maximum number of vertices of our ROIs across participants. After pre-processing, two participants were excluded from analyses due to too much head movement and one participant for not recognizing the majority of the sound stimuli.

Univariate whole-brain and ROI analyses were run on BrainVoyager 22.2 (BrainInnovation). Functional runs were combined and data were entered into a series of general linear models (GLMs), where each sound category was modeled as a separate predictor (two for the animate–inanimate division and four for the intermediate four-category division). A *z*-transform was performed on the resulting beta estimates. Whole brain and univariate ROI results were FDR corrected.

MVPA analyses were run with custom-written MATLAB (version r2015b) scripts, adapted from Smith and Muckli (2010), Muckli et al. (2009), Vetter et al. (2014), and Vetter et al. (2020); see https://github.com/Muckli-lab/MVP-analysis-tool-box). BrainVoyager data was handled through the NeuroElf toolbox (v 1.1) in MATLAB. We estimated beta weights for each sound event in all vertices of each ROI and averaged beta weights across experimental conditions. ROIs were combined across hemispheres to obtain higher statistical power. We applied a *z*-transform to the beta weights before feeding them into a linear support vector machine classification algorithm (Chang and Lin 2001; LIBSVM toolbox, http://www.csie.ntu.edu.tw/∼cjlin/libsvm). We first *z*-normalized the training data (voxel-wise) to lie between −1 and 1 and then applied this normalization to the testing data (Chang and Lin 2001). For each classification, sound event data were labeled according to decoded semantic categories (eg animate/inanimate, or human/animal/vehicle/object etc.). The classifier was then repeatedly trained on two-class classification with the data from 3 runs and tested with the data from the remaining run, in a leave-one-out cross-validation procedure (Pereira et al. 2009). Classification accuracy scores were then averaged across experimental conditions (2, 4, or 12 depending on semantic category level) and across four cross-validation cycles in each individual participant. In the case of the two-category classification, for example, this resulted in individual mean classification accuracies of multiples of 1/8^th^ (= 0.125). These mean classification scores were then averaged across participants for each ROI separately to obtain correct-label group classification accuracy.

To assess statistical significance, we used a permutation analysis, as it is more robust than a *t*-test (Nichols and Holmes 2002; Stelzer et al. 2013). For each participant and each ROI, the classifier was trained and tested 1,000 times with randomized labels for our trials, obtaining distributions with 1,000 classifier performance scores. We computed group means of the randomized distributions and compared them to group means of correct label classifier performance. Then, the group *P*-value was derived as the probability of getting a classifier performance as large as the correct labels’ classifier performance in our randomized labels group distribution. If correct label classification lied in the top 5% of the randomized labels distribution, we concluded significant above chance level decoding at alpha = 0.05 (and accordingly for lower alpha levels). With this method, we computed one group *P*-value per ROI, for each classification performed. Given the robustness of the permutation analysis and independent analyses for each classification (animate versus inanimate, 4-way, and pairwise classifications) and ROI, we did not correct for multiple comparisons across ROIs. However, when comparing pairwise classification accuracies with each other, we applied Bonferroni correction. Effect sizes were computed using Cohen’s d (Cohen 1988).

Whole-brain searchlight analyses were performed on the voxel level with the SearchMight toolbox (Pereira and Botvinick 2011) in MATLAB, using a linear SVM (3-voxel radius). We assessed statistical significance by testing whether the mean classification accuracy across participants was significantly higher than chance at each voxel (ie 0.5 for the animate–inanimate category classification and 0.25 for the four intermediate categories classification). The resulting maps were corrected for multiple comparisons with a cluster threshold correction (*P* < 0.01) calculated with the BrainVoyager Cluster Threshold Plugin tool.

## Results

### MVPA

**Fig. 2:**
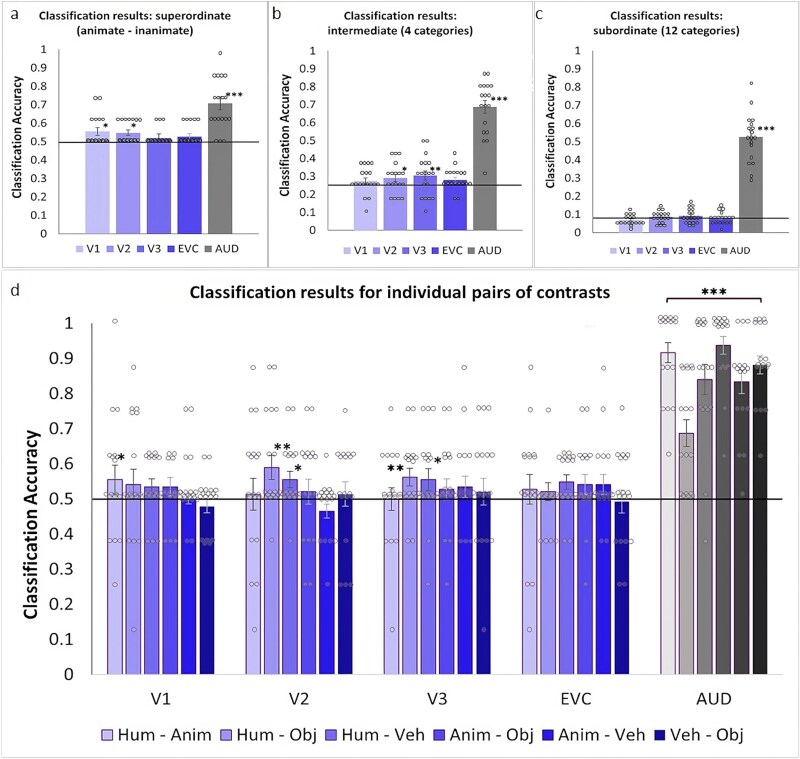
MVPA classification accuracy for (a) animate versus inanimate sounds; (b) humans, animals, vehicles, and objects sounds; (c) the 12 subcategories; and (d) individual pairs of sound categories. Significant above chance classification was derived from permutation analyses across 1,000 iterations of randomized label classification. Solid horizontal lines denote theoretical chance level indistinguishable from permutation derived empirical chance level. Given separate classifications and permutation analyses in a, b, c, and d and each ROI, results were not corrected for multiple comparisons. The bracket over auditory cortex indicates a main effect across all classification pairs. Dots represent individual participants’ data, error bars indicate SEM. ^*^  *P* < 0.05; ^**^ = *P* < 0.01; ^***^  *P* = 0.001.

We first tested whether natural sounds of the super-ordinate categorical level “animate” versus “inanimate” could be distinguished from fMRI activity patterns in early visual cortex. We successfully decoded all animate versus all inanimate sounds significantly above chance, and with large effect sizes, both in V1 and V2 (Fig. 2a; *P* = 0.028, Cohen’s d = 0.93 and *P* = 0.030, Cohen’s d = 0.79 respectively; *P* values determined with permutation analyses).

Next, we decoded sounds belonging to the intermediate categories “humans,” “animals,” “vehicles,” and “objects.” We found that the classifier was able to accurately predict these intermediate sound categories in V2 and V3 with medium effect sizes (Fig. 2b; *P* = 0.028, Cohen’s d = 0.53; *P* = 0.007, Cohen’s d = 0.68). Confusion matrices (Supplementary Fig. S4) showed a tendency that classification worked best for the human and object sound categories in V2 and V3. Classification across all 12 subordinate categories was not successful in early visual cortex (*P* ≥ 0.115), potentially due to classification being performed on the basis of 1/12 of the data (and statistical power) given the 12 subcategories (Fig. 2c, confusion matrices in Supplementary Fig. S4).

Given the successful classification of sounds in early visual areas along the animate–inanimate division, as well as along the division of humans—animals—vehicles—objects, we also ran a pairwise MVPA analysis on each pair of the four categories separately (ie excluding data from the other pairs from the dataset, resulting in 50% of trials and statistical power compared to the animate–inanimate classification). The results showed significant above-chance classification accuracy for the human–animal pair in V1 (*P* = 0.047, Cohen’s d = 0.50), the human–object pair in V2 and V3 (*P* = 0.004, Cohen’s d = 1.04; *P* = 0.018, Cohen’s d = 0.54) and the human–vehicle pair in V2 and V3 (*P* = 0.039, Cohen’s d = 0.57; *P* = 0.040, Cohen’s d = 0.45; Fig. 2d).

In auditory cortex, the same analyses resulted in highly successful above-chance classification accuracy across all sound categories and their pairwise combinations (*P* = 0.001, Cohen’s d > 1).

The significant above-chance classifications in the visual ROIs demonstrated that those pairwise classifications containing human sounds elicited distinguishable activity patterns in at least one early visual cortex region when classified against nonhuman sounds. To directly compare classification accuracies of the pairwise classifications for human and nonhuman sounds across ROIS, we pooled the data into pairs containing human sounds and pairs containing nonhuman sounds. This revealed a significant interaction between ROI and human/nonhuman pairs, with auditory cortex (expectedly) yielding higher classification accuracy than visual regions (repeated-measures analysis of variance (ANOVA), main effect of ROI, F(4,68) = 111.1, *P* < 0.001, main effect of human/nonhuman pairs F(1,17) = 0.25, *P* = 0.62, interaction F(4,68) = 3.99, *P* = 0.011). Post-hoc comparisons showed significantly higher decoding accuracies of human pairs than nonhuman pairs in V2 (*P* = 0.035), and higher decoding accuracies for nonhuman than human pairs in auditory cortex (*P* = 0.006, all Bonferroni corrected).

When including all classification pairs (not categorized into human and nonhuman) and conducting separate ANOVAs for each ROI, classification accuracy did not differ in early visual regions (repeated-measures ANOVA F(5, 85) = 0.412, *P* ≥ 0.116), but differed in auditory cortex (F(5,185) =11.10, *P* < 0.001, Fig. 2d). Post-hoc tests with Bonferroni correction revealed that, in auditory cortex, the human–object classification accuracy was significantly lower than human–animal (*P* < 0.001), animal–object (*P* < 0.001), and vehicle–object (*P* = 0.034) classification.

**Fig. 3:**
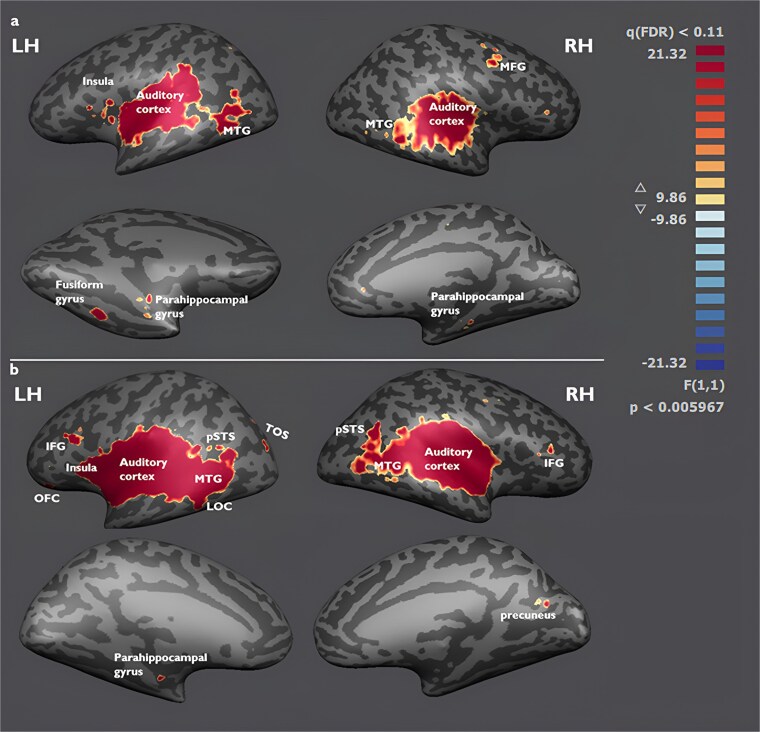
Results of the whole-brain searchlight analysis for (a) the animate–inanimate classification; and (b) the human–animal–vehicle–object classification, cluster threshold corrected. Displayed on an inflated MNI template cortical surface reconstruction.

To investigate whether other brain regions apart from our ROIs could discriminate semantic sound categories, whole-brain searchlight analyses were performed on the voxel level using a linear SVM, implemented with the SearchMight toolbox (Pereira and Botvinick 2011). This analysis revealed above-chance classification for animate versus inanimate sounds in the auditory cortex bilaterally, as well as in the middle temporal gyrus, right middle frontal gyrus, left fusiform gyrus, and bilateral parahippocampal gyrus (Fig. 3a). As for the four-way classification, auditory cortex predictably showed above-chance decoding bilaterally once again, as well as bilateral middle temporal gyrus (MTG) and left parahippocampal gyrus. Additionally, bilateral inferior frontal gyrus (IFG), bilateral posterior superior temporal sulcus (pSTS), left lateral occipital complex (LOC), right precuneus and left transverse occipital sulcus (TOS) were also identified as regions with above-chance classification of the four-sound categories (Fig. 3b). Note that whole-brain searchlight MVPA results require correction for multiple comparisons across the whole brain, resulting in less sensitivity than ROI MVPA analyses, and early visual cortex not surviving this correction on the whole-brain level.

### Whole-brain univariate analyses

**Fig. 4:**
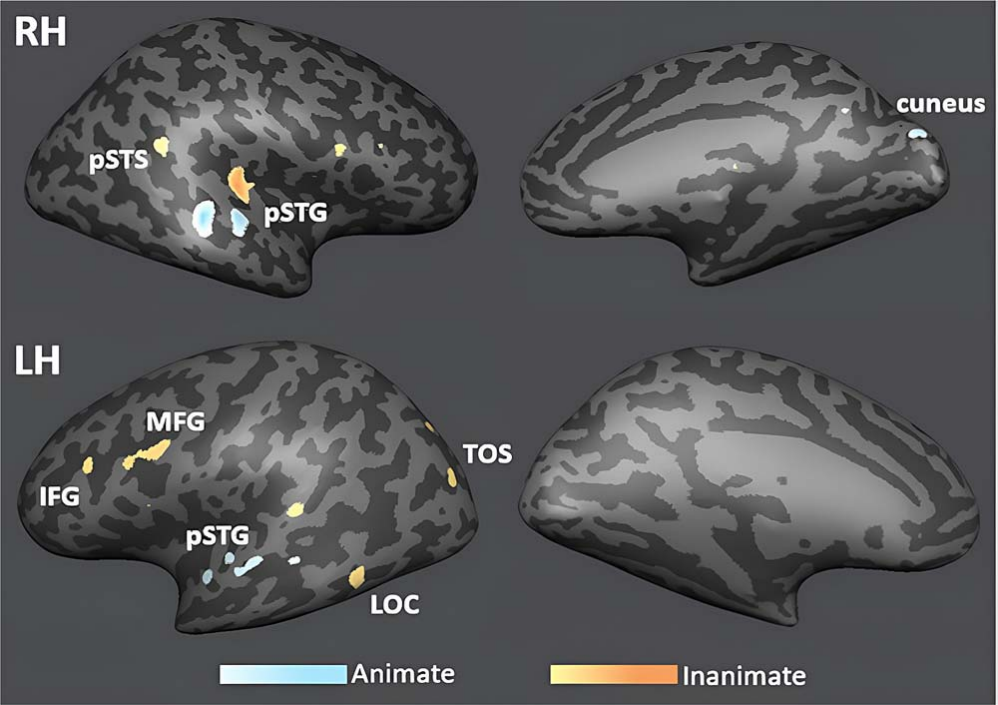
GLM analysis results of the inanimate > animate contrast projected onto an inflated MNI template cortical surface reconstruction with *P* = 0.01 (FDR corrected), with warm colors representing significant activation for inanimate sounds and cold colors indicating significant activation for animate sounds.

We also performed a series of GLM analyses. A whole-brain GLM with the contrasts inanimate versus animate revealed that animate sounds activated more lateral regions of the auditory cortex, whereas inanimate sounds activated more medial regions of auditory cortex and a more widespread network including the left TOS, IFG, MFG, and LOC (Fig. 4).

**Fig. 5:**
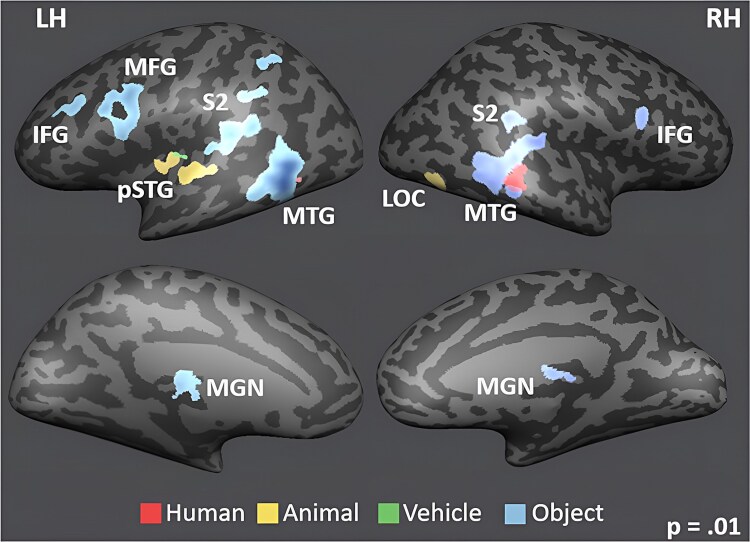
GLM analyses results of significant activations for each of the four categories (human, animal, vehicle, and object) against the other three categories (color coded as per legend). Contrasts are projected onto an inflated MNI template cortical surface reconstruction with *P* = 0.01 (FDR corrected).

Comparing activation of each sound category “humans,” “animals,” “vehicles,” and “objects” against the other three did not produce significant differences in early visual cortex but revealed a distinct pattern for each category across the temporal brain, particularly in left pSTG and right LOC for animals and a larger network of regions including bilateral IFG and MTG, and left MFG for objects (Fig. 5).

### Univariate ROI analyses

**Fig. 6:**
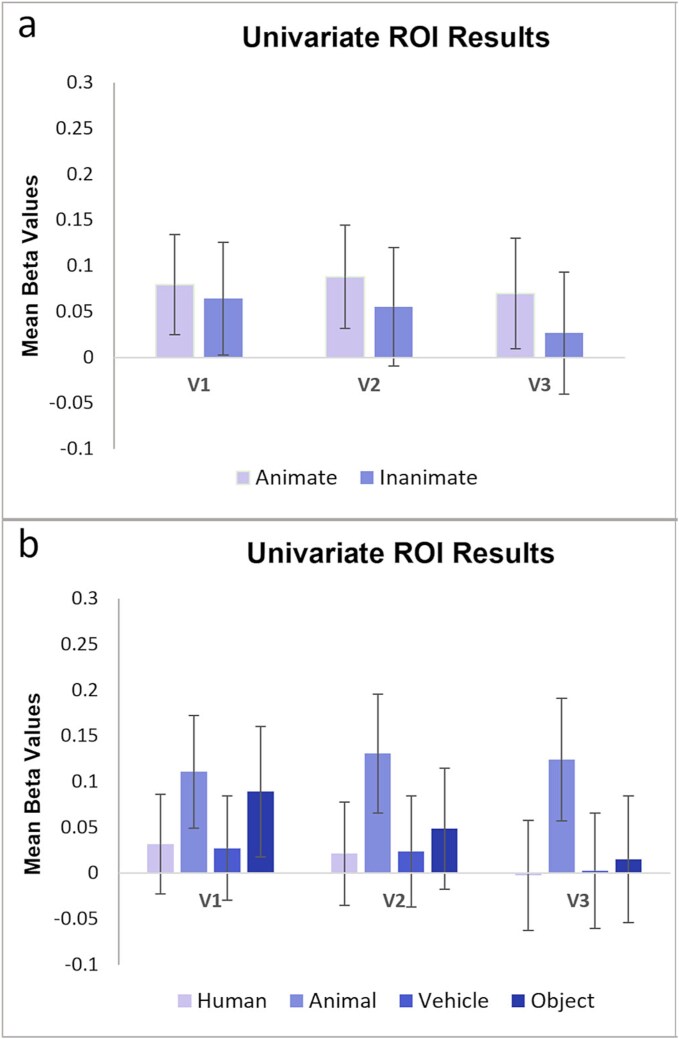
Univariate ROI activity levels for the (a) two-way, and (b) four-way categorization. Mean beta values are plotted for each sound condition in V1, V2, and V3, relative to baseline. Error bars indicate SEM.

To investigate whether our MVPA results in our ROIs V1, V2, and V3 were driven by univariate activity differences, we conducted a GLM analysis in these ROIs contrasting activity of the different sound categories against baseline. Beta values for each ROI were averaged across hemispheres for each of our sound category distinctions and then across participants (Fig. 6).

We did not find an effect of animacy when comparing univariate activity levels for animate versus inanimate sounds across V1, V2, and V3 (repeated measures ANOVA (main effect of sound type F(1,17) = 1.88; *P* = 0.188; main effect of visual areas F(2,34) = 0.42; *P* = 0.564; interaction F(2,34) = 2.10; *P* = 0.159). The mean beta values for these two categories were not significantly different from baseline (*t*(17) < 1.56, *P* > 0.465, FDR corrected). We also did not find a main effect of category when comparing activity levels for the four categories (main effect of sound type F(3,51) = 2.63; *P* = 0.060; main effect of visual areas F(2,34) = 0.71; *P* = 0.428; interaction F(6,102) = 2.67; *P* = 0.070). Comparing the four categories’ mean activations against baseline for all ROIs revealed no significant difference (*t*(17) < 2.21, *P* > 0.308, FDR corrected).

### Correlation analyses with vividness of imagery

**Fig. 7:**
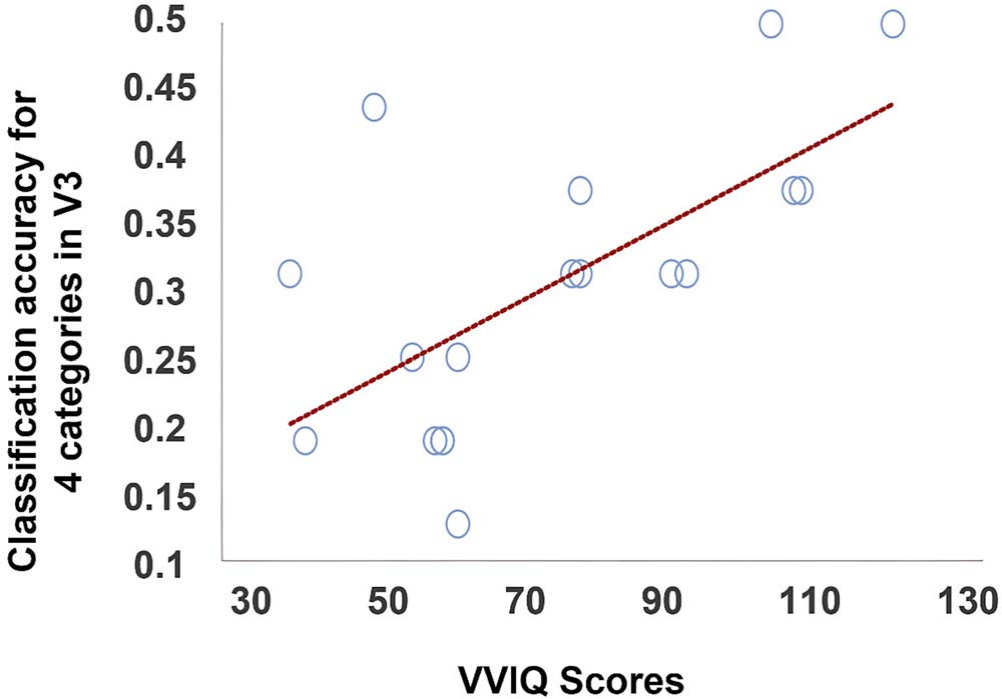
Individual scores from the vividness of visual imagery questionnaire plotted as a function of sound classification accuracy for the four-category classification in V3. Note that high VVIQ scores denote less vivid visual imagery.

To investigate whether MVPA classification of sounds in early visual cortex may have been driven by visual mental imagery, we correlated the scores from the VVIQ questionnaires with classification accuracy scores for all ROIs in early visual cortex and auditory cortex using Pearson’s correlation coefficient. Higher VVIQ scores, corresponding to less vivid visual imagery, positively correlated with four-category classification accuracy in V3 (*r*(17) = 0.658, *P* = 0.003; FDR-corrected *P* = 0.045), but did not correlate in either of the other ROIS (V1, V2, EVC, and auditory cortex), nor with the two-category classification accuracy in any ROI (r (17) < 0.490, FDR-corrected *P* > 0.195). The positive correlation of VVIQ scores and classification accuracy indicated that participants with less vivid visual imagery displayed more distinguishable activity patterns in V3 than participants with highly vivid visual imagery (Fig. 7). This adds a small piece of evidence against visual imagery having strongly driven successful sound classification, at least in V3.

### Sound stimuli analyses

To test whether sound categories differed in their acoustic features, we derived power spectrum density (PSD, a combination of amplitude and frequency). PSD of each sound as well as their averages for the human, animal, object, and vehicle category are plotted in Supplementary Fig. S5. Comparing average PSDs across the four different categories revealed significant main effects of sound category, but only in selected narrow frequency ranges: 6,890 to 7,407 Hz, 7,751 to 8,096 Hz, 9,474 to 9,991 Hz, 10,163 to 10,335 Hz, and 10,508 to 10,852 Hz (ANOVA ([HUM vs ANM vs OBJ vs VEH], F(3,32) > 5.50, *P* < 0.05, FDR corrected; see Supplementary Fig. S5). None of the post-hoc comparisons contrasting each category with another survived FDR-correction (*P* > 0.05).

We also computed the harmonics-to-noise ratio (HNR), representing the degree of acoustic periodicity, according to Lewis et al. (2005). Comparing maximum HNR across the human, animal, object, and vehicle sounds (plotted in Supplementary Fig. S6) resulted in a main effect of sound category (F(3, 32) = 3.57, *P* = 0.025). While the vehicle sounds seem to exhibit lower max HNR than the other sound categories, none of the post-hoc comparisons survived FDR correction (VEH vs HUM: *t*(16) = 2.37, p_FDR_ = 0.062; VEH vs ANM: *t*(16) = 2.65, p_FDR_ = 0.053; VEH vs OBJ: *t*(16) = 2.75, p_FDR_ = 0.053).

We also derived amplitude envelopes and analyzed attack (the relative time to reach the maximum amplitude from the start of the sound) for the first local normalized amplitude peak of each sound. Comparing attack across sound categories (plotted in Supplementary Fig. S7) revealed no main effect of category (F(3, 32) = 0.58, *P* = 0.63).

## Discussion

The current study investigated which type of auditory semantic information content is represented in the neural activity patterns of early visual cortex. Despite early visual cortex being associated mostly with visual functions, we found that natural sounds belonging to several different semantic categories can be decoded from fMRI activity patterns in early visual cortex, in the absence of visual input. Sounds belonging to superordinate semantic categories like “animate” and “inanimate” could be decoded in early visual cortex (V1 and V2), replicating and extending our previous results (Vetter et al. 2014; Vetter et al. 2020) with a much larger variety of individual sound stimuli per category and demonstrating categorical sound decoding also in V1. In addition, we show here that sounds belonging to more specific categories such as humans, animals, vehicles, and objects can also be decoded from activity patterns in early visual cortex. This latter novel finding suggests that the information transferred from auditory cortex to early visual cortex carries semantic information content that goes beyond the animacy/inanimacy distinction, following the more fine-grained categorical distinction of human/animal/vehicle/object. This is evidence for fine-grained categorical sound distinction in early visual cortex in blindfolded sighted participants, and it is nicely mirrored by sound decoding results in blind participants, both in early visual cortex and ventral temporal cortex (Van den Hurk et al. 2017; Mattioni et al. 2020). The representation of semantic sound categories in early visual cortex reveals the type of information content that is communicated from auditory cortex to early visual cortex via feedback and thus adds an important puzzle piece to revealing the information content of top-down feedback to early visual cortex (eg Petro et al. 2014; Petro et al. 2017). Clarifying the information content of feedback is critical for understanding predictive processes in vision (eg Bar 2007; Friston 2010) as well as understanding early visual cortex function beyond simple visual feature processing (eg Roelfsema and de Lange, 2016).

Interestingly, our classifier was also able to distinguish between some individual pairs of sound categories when the pair included the “human” category (human–animal in V1, human–object and human–vehicle in V2 and V3). Directly comparing decoding accuracies across pairwise classifications confirmed that classification pairs containing human sounds could be decoded better in V2. These results suggest that human-produced sounds are represented in the visual brain, even down to early visual cortex, in a way that makes them distinguishable from other sound categories. This opens the possibility that the brain may treat stimuli from the human category more distinctly than other semantic categories, even when sensory information comes from audition and is represented at the earliest stages of visual processing. Again, these results mirror findings in congenitally blind and sighted individuals showing that early visual cortex distinguishes meaningful from nonmeaningful human speech stimuli (Musz et al. 2022: Seydell-Greenwald et al. 2023) and is activated by human action-related sounds in the blind (Lewis et al. 2011).

In auditory cortex, the classifier performance was well above chance. This was expected given that auditory cortex is specialized in distinguishing sounds and given that different animate and inanimate sounds activate distinct areas of auditory cortex even when applying less sensitive univariate analyses (eg Lewis et al. 2005; Engel et al. 2009; Brefczynski-Lewis and Lewis 2017), confirmed by our univariate whole-brain results (Figs. 4 and 5). However, classifier performance in auditory cortex for the human–object classification was significantly worse than for the other pairs (Fig. 2d). A possible explanation could be that the samples we chose for object sounds mostly came from human-produced actions (eg spoon stirring in a teacup, hammering, etc.). This was partly unavoidable as objects usually only emit sounds when interacted with by humans, potentially blurring the categorical distinction between human and object sounds. Consequently, this blurred categorical distinction could have led to similar representations in auditory cortex (potentially also in V2, see confusion matrices in Supplementary Fig. S4), resulting in less distinct representations between human and object sounds and decreased classification accuracy.

Why may human-produced stimuli be more distinctly represented in early visual cortex than other categories? It is possible that the brain devotes a lot of computational power to interpreting and representing human stimuli due to their high relevance for social interaction. Examples come from the literature on face and speech perception which indicate how, for humans, conspecifics are better and preferentially detected than other living beings (Pascalis et al. 2002; Vouloumanos et al. 2010). Humans are able to extract a variety of information from human faces and voices, ranging from identity to personality, to emotion and mental state (McAleer et al. 2014; Maguinness et al. 2018; Oruc et al. 2019). Thus, the current findings on the ability of early visual cortex to distinguish human sounds fit nicely with the literature that supports tuning for conspecifics in the human brain. A potential explanation for our results is that the brain prioritizes the information about other humans in the environment and sends this information via feedback to several brain areas, including early visual cortex, such that it is optimally prepared to precisely identify and predict human stimuli most relevant for social interaction.

The distinctiveness of neural representations of semantic sound categories in early visual cortex is unlikely to be driven by a similarity of the acoustic features of our sound samples in one category compared to the other categories, since each category contained several sound exemplars with a wide range of acoustic frequency patterns (see sound amplitude profiles, amplitude envelopes, and spectrograms in Supplementary  Figs. S1 to S3). While average power spectral density profiles (Supplementary Fig. S5) partly differed across sound categories, this was restricted to a few selected narrow frequency ranges, and no PSD profile of one category significantly differed from another category. Similarly, the marginally lower HNR for the vehicle sounds (Supplementary Fig. S6, not surviving FDR correction) was not reflected in the pairwise classifications in early visual cortex (Fig. 2d).

Thus, while acoustic differences may underlie successful sound classification in auditory cortex, it is unlikely that they caused successful classification in early visual cortex, particularly in the pairwise classifications. Instead, our classification results in visual cortex must have been driven by the information content that is both shared across all the different sound exemplars of one category and distinct from the shared information content of the other categories. Thus, the classifier was able to generalize across many different sound exemplars within a category; otherwise it would not have been able to distinguish one category from another above chance. For the same reason, successful classification in visual cortex was unlikely to be driven by individual sound exemplars eliciting more attention, arousal, emotional response or eye or body movements. If any of these factors drove classification in visual cortex, they must have done so consistently across the majority of sounds within a specific category and not in the other categories; otherwise the classifier would not have succeeded. Also, these factors should have increased univariate BOLD signal in the visual ROIs (eg Smith et al. 2006), but univariate signal in early visual cortex did neither significantly differ from baseline nor across sound categories (Fig. 6). Therefore, the classifier must have picked up on the small and spatially distributed activity differences within each ROI and extracted the shared information content across all activity patterns from one category. The case is different for auditory cortex, here we did find univariate modulation of BOLD signal in response to different sound categories (Figs. 4 and 5), and thus classification accuracy may have likely been boosted by these univariate signal differences.

Following the same logic, our classification results in visual cortex are unlikely to be entirely driven by activity patterns potentially evoked by sound-induced visual imagery—the potential visual images evoked by the different sounds would differ substantially in visual features across exemplars within a category (eg finger snapping and footsteps; horse galloping; and seagull). If visual imagery, in terms of a re-activation of a visual perception-like representation, had driven our positive classification results, then all individual sound exemplars should have elicited highly distinguishable activity patterns in early visual cortex due to the differential low-level visual features of the mental imagery representations. Thus, our classifier should have performed with much higher accuracy, distinguishing all activity patterns from each other, but not being able to generalize across activity patterns elicited by different sounds. Instead, what we find is that the classifier can generalize across activity patterns elicited by many different sounds from one semantic category (in the current study 18 exemplars from the animate category, 18 from the inanimate category, and 9 of each of the intermediate categories). That is, what we decode here is the shared information content of all stimuli within one category that is distinct from the shared information of the other categories (ie animacy/inanimacy or humanness/animalness/objectness/vehicleness). Our results therefore indicate categorical and semantic information representation in early visual cortex, not sound exemplar- or mental image-specific information. The current category-specific classifications extend and mirror results from our earlier study (Vetter et al. 2014, Exp. 5), in which we had explored the role of visual imagery in more detail with several fMRI experiments. In another earlier study, we had directly addressed the potential visual imagery contribution by testing participants who are blind from birth and entirely lack visual imagery (Vetter et al. 2020). In early “visual” cortex of these congenitally blind individuals, sounds along the categories human–animal–inanimate can be decoded with even higher accuracy than in blindfolded sighted participants. Thus, even if visual imagery may contribute to the successful classification of sounds in early visual cortex of the sighted, then it should boost classification accuracy in the sighted (making it easier for the classifier to distinguish between activity patterns), when in fact, we found the opposite result pattern (lower classification accuracy in the sighted than the blind; Vetter et al. 2020). Furthermore, even in aphantasic individuals who have much reduced or absent visual mental imagery abilities, sound decoding in early visual cortex is reduced, but still successful (Cabbai et al. 2024; Montabes de la Cruz et al. 2024). Thus, even in the absence of visual mental imagery, either due to aphantasia or congenital blindness, sound information is still represented in early visual cortex activity patterns and thus a simple visual imagery account is unlikely to entirely explain successful sound classification in sighted participants.

Also, in the current study, the absence of significant above or below baseline univariate activation and the absence of univariate activity differences across sound categories in early visual cortex (Fig. 6) speaks against sound classification being driven by visual imagery (or attention) strongly activating or deactivating early visual cortex. Instead, as mentioned above, the classifier picked up on the differential spatial distribution of small activity differences across voxels/vertices in each sound category which indicates differential neural representations and differential information content (eg Pereira and Botvinick 2011; Haxby et al. 2014). While correlation results need to be interpreted with caution, the finding that participants with more vivid visual imagery showed lower four-way classification accuracy in V3 is another small piece of evidence that visual imagery is unlikely to have strongly driven classification. Thus, potential imagery during sound presentation may have negatively interfered with sound classification in V3 and at least did not boost classifier performance.

Our findings from the whole-brain searchlight analysis revealed that a network of regions beyond auditory cortex is sensitive to the categorical distinction between animate and inanimate sounds and human, animal, vehicle and object sounds. Above-chance four-way classification suggests that category-specific information about sounds is also represented in regions implicated in multisensory integration such as the precuneus (Ripp et al. 2018; Park and Kayser 2019), or integration of semantically matching audio–visual stimuli, such as the pSTS (Beauchamp et al. 2004; Doehrmann and Naumer 2008; Naumer et al. 2011; Plank et al. 2011). While our results do not allow conclusions about the exact brain pathways auditory information travels to reach visual cortex, these multisensory regions might serve as mediators between sensory areas and potentially relaying information from auditory to early visual cortex (see also Werner and Noppeney 2010). Both searchlight analyses revealed above chance classification in the insula which is often active during auditory stimulation (Uddin et al. 2017) and, more importantly, active during experience of perceptually related audiovisual stimuli (Naghavi et al. 2007). Animate and inanimate sound decoding was also successful in fusiform gyrus and parahippocampal gyrus, regions of the ventrotemporal cortex involved in higher level visual processing. Fusiform gyrus plays a crucial role in face recognition (Kanwisher et al. 1997; Ganel et al. 2005, Tsao et al. 2006), while the parahippocampal gyrus is active during scene recognition (Epstein and Kanwisher 1998). Their domain selectivity has been reported to exist beyond visual stimulation, as the FFA and PPA of congenitally blind people has been shown to robustly respond to haptic exploration of 3D faces (Ratan Murty et al. 2020) and scenes (Wolbers et al. 2011). This, together with our findings from auditory stimulation, suggests that these regions may integrate information from different sensory modalities for the recognition of human and nonhuman stimuli.

Interestingly, LOC was also able to significantly distinguish between sound categories in our whole-brain searchlight four-way classification. LOC is involved in visual object recognition (Grill-Spector et al. 1999), and has been found to be implicated in audiovisual integration and memory of objects (Amedi et al. 2005; Murray et al. 2005; Doehrmann et al. 2010; Giovannelli et al. 2016), during visual-to-auditory sensory substitution (Amedi et al. 2007) and when objects are recognized haptically (Amedi et al. 2001). While these earlier studies using standard univariate analyses failed to show LOC activity with solely auditory stimulation, we show here with more sensitive MVPA that LOC can in fact distinguish between auditory categories.

What is the purpose of category-specific information from audition being fed back to early visual cortex? Both touch and sound have been shown to enhance activity in early visual cortex (Macaluso et al. 2000; Martuzzi et al. 2007; Ramos-Estebanez et al. 2007), so a potential way in which early visual cortex may employ auditory feedback is to predict incoming visual information (Bar 2007; Friston 2010; Petro et al. 2017) and, eventually, enhance visual perception. On the behavioral level, semantic auditory information has been shown to facilitate visual object and scene recognition when visual information is noisy (Chen and Spence 2010; Chen and Spence 2011; Lu et al. 2018; Williams et al. 2022; Williams and Störmer 2024), or made ambiguous during binocular rivalry (Chen et al. 2011b) or during continuous flash suppression (Ioannucci and Vetter 2025). Thus, auditory feedback is likely to be particularly important for precise visual perception when visual input is unclear. Another option is that auditory information is represented in early visual cortex for the potential purpose of audio–visual interaction or integration (Murray et al. 2016), but our data do not allow conclusions about either interaction or integration as we deliberately omitted visual stimulation.

To conclude, our results provide further evidence that early visual cortex function is not restricted to the processing of low-level visual features from retinal input, but also for the representation of higher level features from other sensory modalities. This evidence is critical for clarifying the information content of top-down feedback to early visual cortex and its potential role in visual prediction and perception (Vetter et al. 2024).

## Supplementary Material

PollicinaMuellerDaltonVetter_SupplementalMaterial_finalVersion_bhaf208

## Data Availability

The data that support the findings of this study is openly available and stored on OpenNeuro at https://openneuro.org/datasets/ds006486.
