# Supplementary material for: Decoding semantic sound categories in early visual cortex
Source: Cereb Cortex. 2025 Aug 2;35(8):bhaf208. doi: 10.1093/cercor/bhaf208 (PMC12317377; doi:10.1093/cercor/bhaf208)
Supplement: PollicinaMuellerDaltonVetter_SupplementalMaterial_finalVersion_bhaf208 [file pollicinamuellerdaltonvetter_supplementalmaterial_finalversion_bhaf208.docx]

**Supplemental Materials
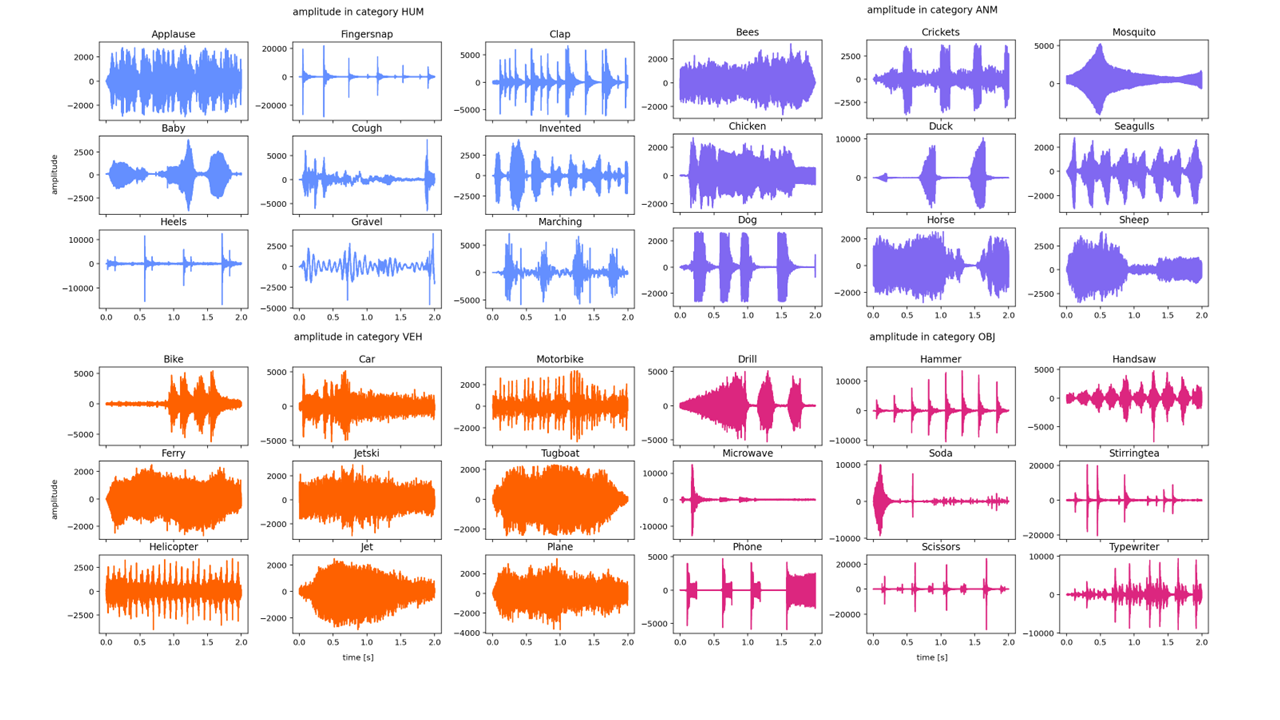
**

***Figure S1.*** *Amplitude profiles of each of the 36 sounds used in the fMRI experiment, categorized into human (blue), animal (purples), vehicle (orange) and object (magenta) sounds.*

*
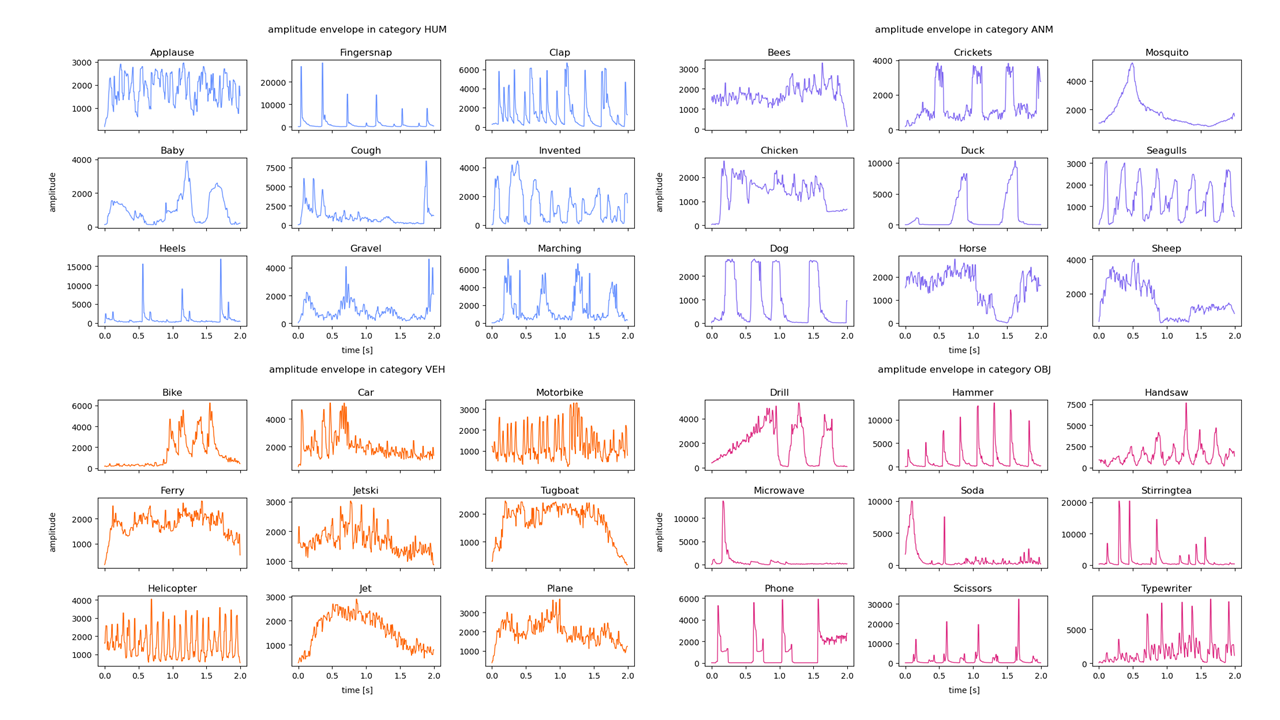
*

***Figure S2.*** *Amplitude envelopes of each of the 36 sounds. Categorisation as in Fig. S1.*


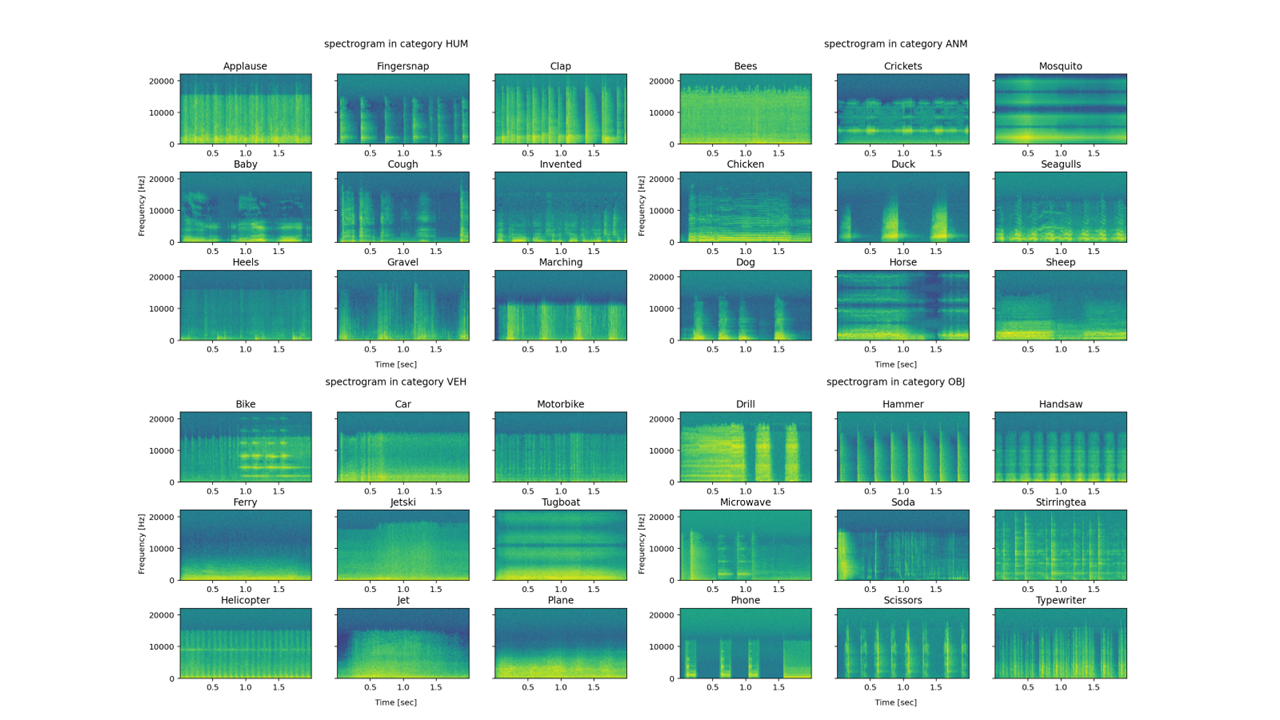


***Figure S3.*** *Spectrograms of each of the 36 sounds. Categorisation as in Fig. S1.*

*
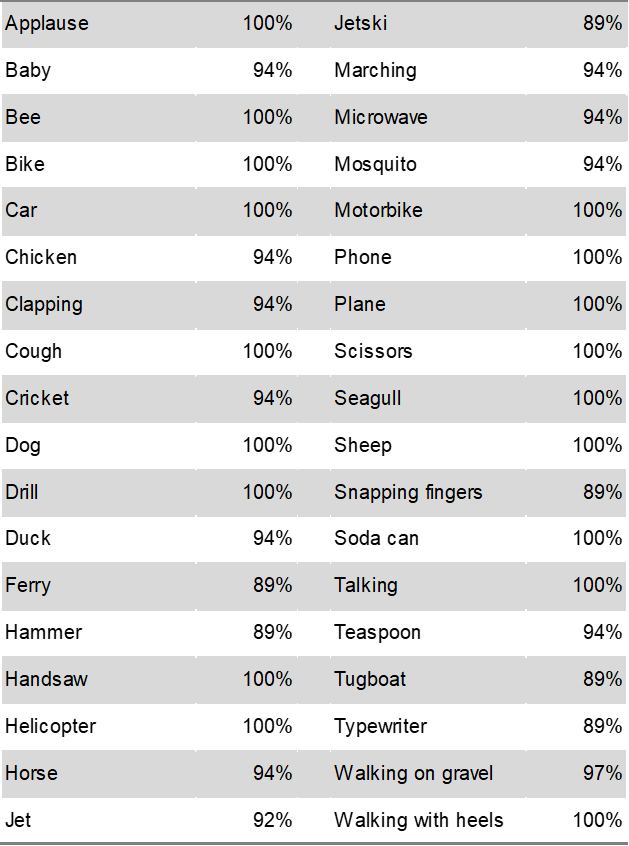
*

***Table S1.*** *Percentage of participants accurately identifying each sound post-scanning.*

**Region Mean Min Max**

**AUD** 1,133 333 2,386

**V1** 2,578 1,526 3,901

**V2** 2,679 1,398 3,876

**V3** 2,192 1,442 3,390

**EVC** 7,173 4,309 8,817

***Table S2:*** *Mean, minimum and maximum number of vertices of each individually mapped Region of Interest across participants.*


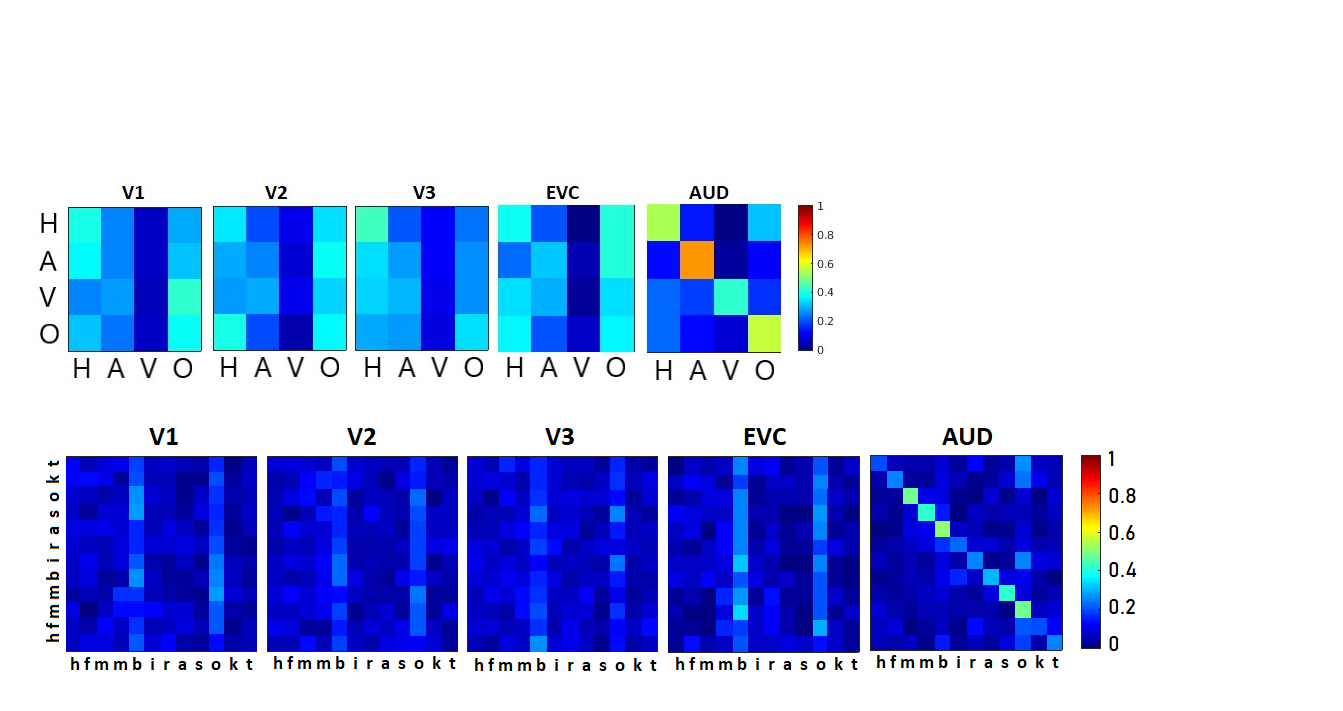


***Figure S4.*** *Confusion matrices of the classifier predictions for the 4-way classifications (upper panel) and the 12-way classifications (lower panel). Columns of confusion matrices indicate the presented sounds and rows indicate the classifier’s prediction for this sound. Colours indicate the classifier’s prediction accuracy for each combination of presented and predicted sound. Note that chance level differs for the 4-way classification (0.25) and the 12-way classification (0.083). Sounds in the 4-way classifications were H= human, A= animal, V= vehicles and O=objects. Sounds in the 12-classifcations were h = hand, f = foot, m = mouth (human category); m = mammals, b = birds, i = insects (animal category), r = road vehicles, a = air vehicles, s = ships (vehicle category); o = office utensils, k= kitchen utensils, t =tools (object category).*


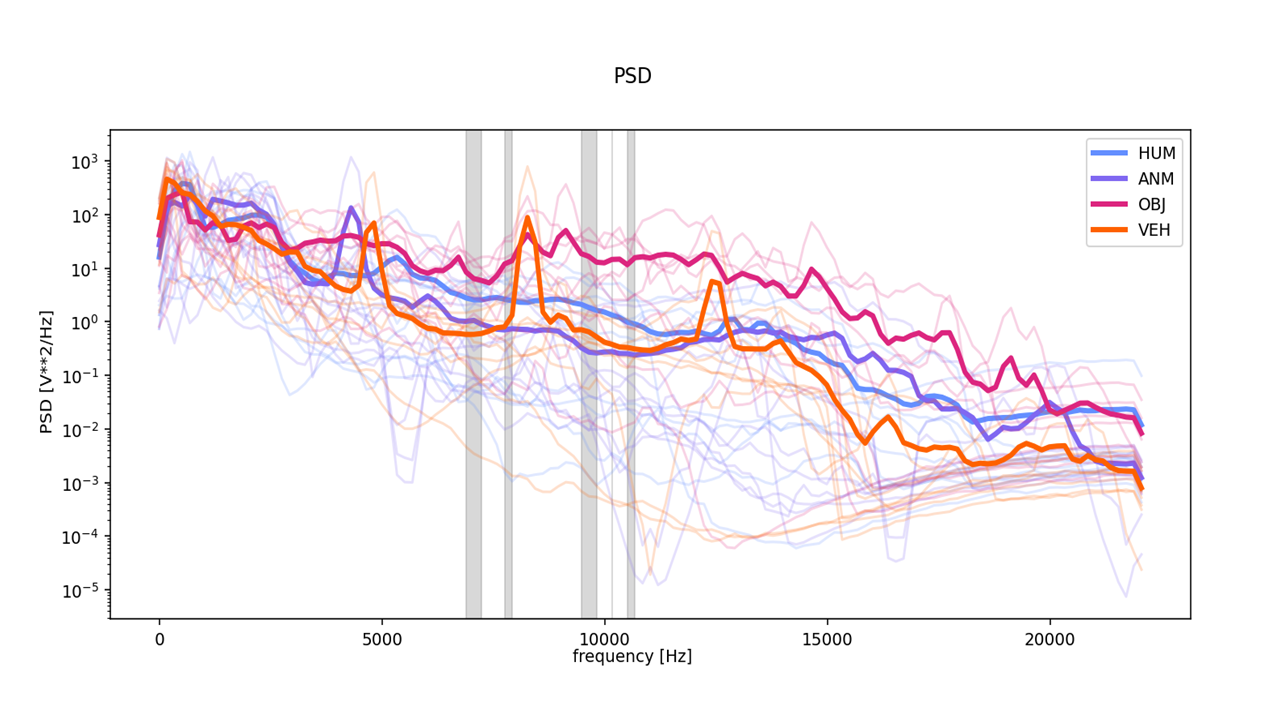


***Figure S5.*** *Power spectral density (PSD) profiles of each of the 36 sounds (light colours) used in the fMRI experiment. Bold lines (dark colours) represent average PSDs across sounds in each category (see colour legend). Shaded grey areas indicate frequency ranges where there was a main effect of category (ANOVA [HUM vs ANM vs OBJ vs VEH], pFDR < .05). None of the post-hoc comparisons contrasting each category with another survived FDR correction (p > .05).*

*
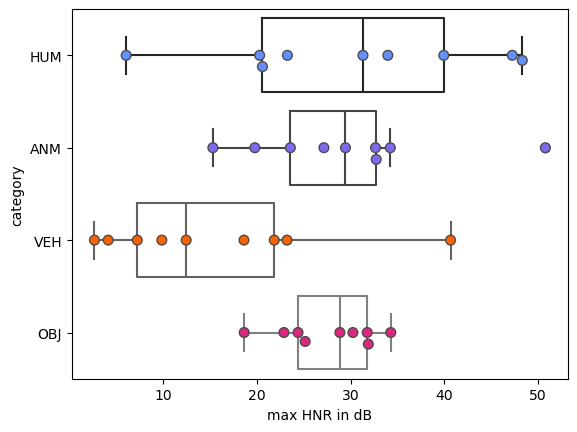
*

***Figure S6****. Maximum Harmonics-to-Noise Ratios plotted as a function of sound category. An ANOVA revealed a main effect of sound category (F (3, 32) = 3.57, p = .025). While the vehicle sounds seem to exhibit lower max HNR than the other sound categories, none of the post-hoc comparisons survived FDR correction (**VEH vs HUM: t (16) = 2.37, p_FDR_ = .062; VEH vs ANM: t (16) = 2.65, p_FDR_ = .053; VEH vs OBJ: t (16) = 2.75, p_FDR_ = .053)*.

*
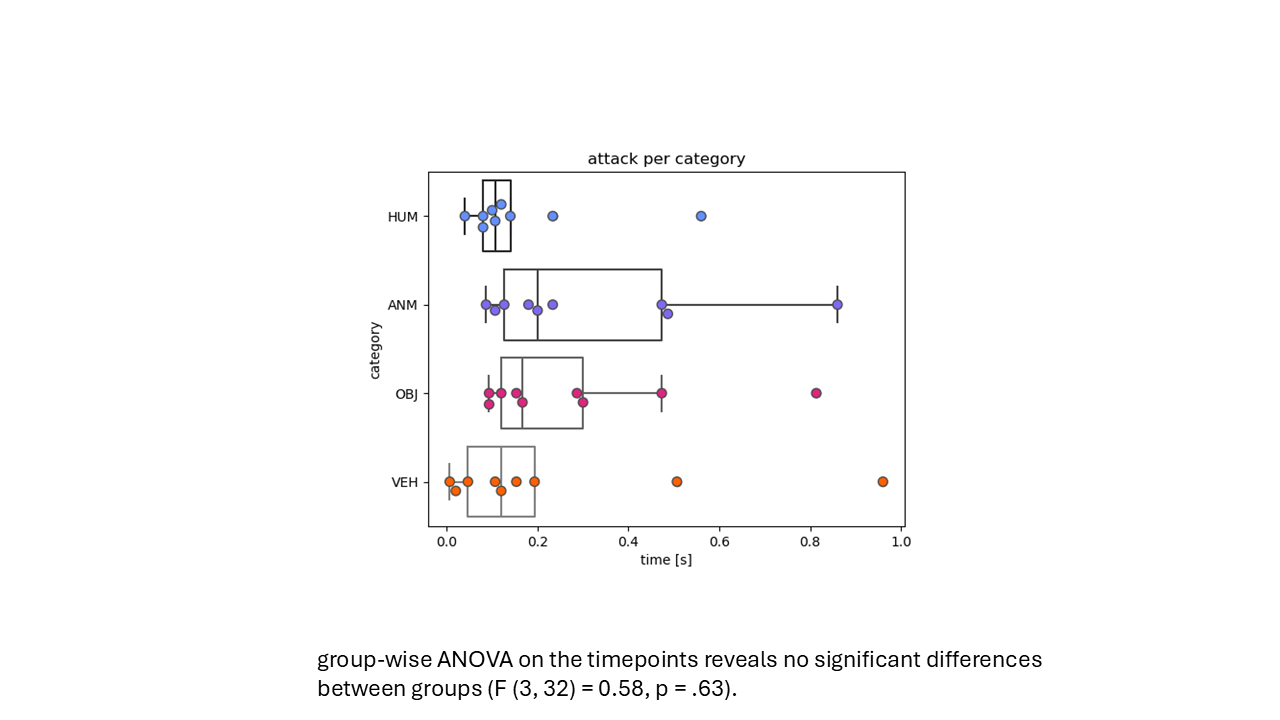
*

***Figure S7****. Attack per sound category. Attack is defined here as the first timepoint where the preceding timepoint as well as all 20 succeeding timepoints are smaller in amplitude, and the normalised amplitude exceeds 0.3. Comparing attack across sound categories showed no main effect of category (F (3, 32) = 0.58, p = .63).*
